# Case Report: Marfan syndrome complicated with obturator hernia

**DOI:** 10.3389/fsurg.2026.1797226

**Published:** 2026-04-21

**Authors:** Danning Zhang, Zining Chen, Xiaolong He, Qingbo Feng

**Affiliations:** 1 Zunyi Medical University, Zunyi, Guizhou, China; 2 Department of General Surgery, Digestive Disease Hospital, Affiliated Hospital of Zunyi Medical University, Zunyi, Guizhou, China; 3 Guizhou Provincial Key Laboratory of Digestive System Diseases, Zunyi, Guizhou, China

**Keywords:** case report, laparoscopic surgery, Marfan syndrome, obturator hernia, TAPP

## Abstract

**Background:**

Obturator hernia is an extremely rare subtype of abdominal wall hernia, typically occurring in elderly female with a history of chronic increased intra-abdominal pressure or multiple pregnancies. However, it is rarely encountered in young individuals, which may lead to diagnostic oversight. Here, we report a case of a young female with Marfan syndrome who developed a left obturator hernia

**Patient presentation:**

A young female with Marfan syndrome and pectus excavatum presented with 6 h of persistent severe left inguinal pain.

**Diagnostic process:**

Physical examination revealed a 2 cm × 2 cm localized swelling in the upper medial aspect of the left thigh, medial to the inguinal ligament. Left Howship-Romberg sign (+). Computed tomography (CT) examination indicated pectus excavatum, cardiomegaly, and a left obturator hernia.

**Intervention:**

Based on these findings, laparoscopic preperitoneal inguinal hernia mesh repair was performed.

**Outcome:**

The patient was discharged on postoperative day 3 and followed up for 14 months. Recovery was uneventful with no complications.

**Conclusion:**

This case highlights the importance of differential diagnostic thinking for hernias in patients with connective tissue diseases.

## 1 Introduction

Obturator hernia is an extremely rare external abdominal hernia that typically occurs in elderly multiparous females. When intra-abdominal pressure elevated, abdominal contents may protrude through the obturator canal (1, 2). Marfan syndrome is an autosomal dominant hereditary connective tissue disorder caused by mutations in the FBN1 gene, resulting in skeletal abnormalities, lens dislocation, pneumothorax, and cardiovascular disorders (3).

Given the rarity of obturator hernia in young individuals and the fact that recurrent hernia and incisional hernia are minor diagnostic criteria for Marfan syndrome, obturator hernia in patients with Marfan syndrome is extremely rare (4). Herein, we present a 26-year-old female patient with Marfan syndrome who was diagnosed with obturator hernia.

## 2 Case presentation

### 2.1 Patient information

A 26-year-old female patient was admitted to the emergency department complaining of persistent severe pain in the left inguinal region for 6 h. The pain was constant, non-radiating initially but later extended to the medial aspect of the left thigh, and was not accompanied by associated symptoms such as nausea, vomiting, abdominal distension, fever, or changes in bowel and bladder function—symptoms that are often present in typical obturator hernia cases with intestinal obstruction.

Her past medical history was significant: she underwent aortic valve replacement surgery 6 years prior due to congenital heart disease, a common cardiovascular complication of Marfan syndrome. Intraoperative blood transfusion was performed. and postoperative recovery was uneventful. The patient has been taking oral warfarin postoperatively with regular coagulation function tests, and the dosage was adjusted accordingly. 2 years earlier, she underwent a cesarean section and postoperative recovery was uneventful. Oral warfarin was suspended during admission for labor and resumed after delivery, and has been continued ever since. Otherwise, the patient denied a history of hypertension, diabetes mellitus, coronary heart disease, and denied a history of infectious diseases such as hepatitis and tuberculosis. The patient reported a history of allergy to clindamycin and Chinese yam. Family history was unknown.

### 2.2 Diagnostic assessment

Physical examination yielded characteristic findings: the patient appeared acutely distressed. Tenderness, localized rebound tenderness and muscular rigidity were noted in the left abdomen. Palpation of the deep medial area of the left inguinal ligament, just superior to the medial aspect of the left thigh, revealed a localized swollen mass approximately 2 cm × 2 cm in size. The mass was firm in texture, with ill-defined borders and marked tenderness (+); it was irreducible by gentle manual compression, and no obvious impulse was elicited during coughing or Valsalva maneuver (which increases intra-abdominal pressure), suggesting a non-reducible hernia. Notably, a positive left-sided Howship-Romberg sign was detected—an important pathognomonic sign for obturator hernia, characterized by exacerbated pain when the left hip is flexed, abducted, and externally rotated, due to compression of the obturator nerve by the hernia sac.

**Figure 1:**
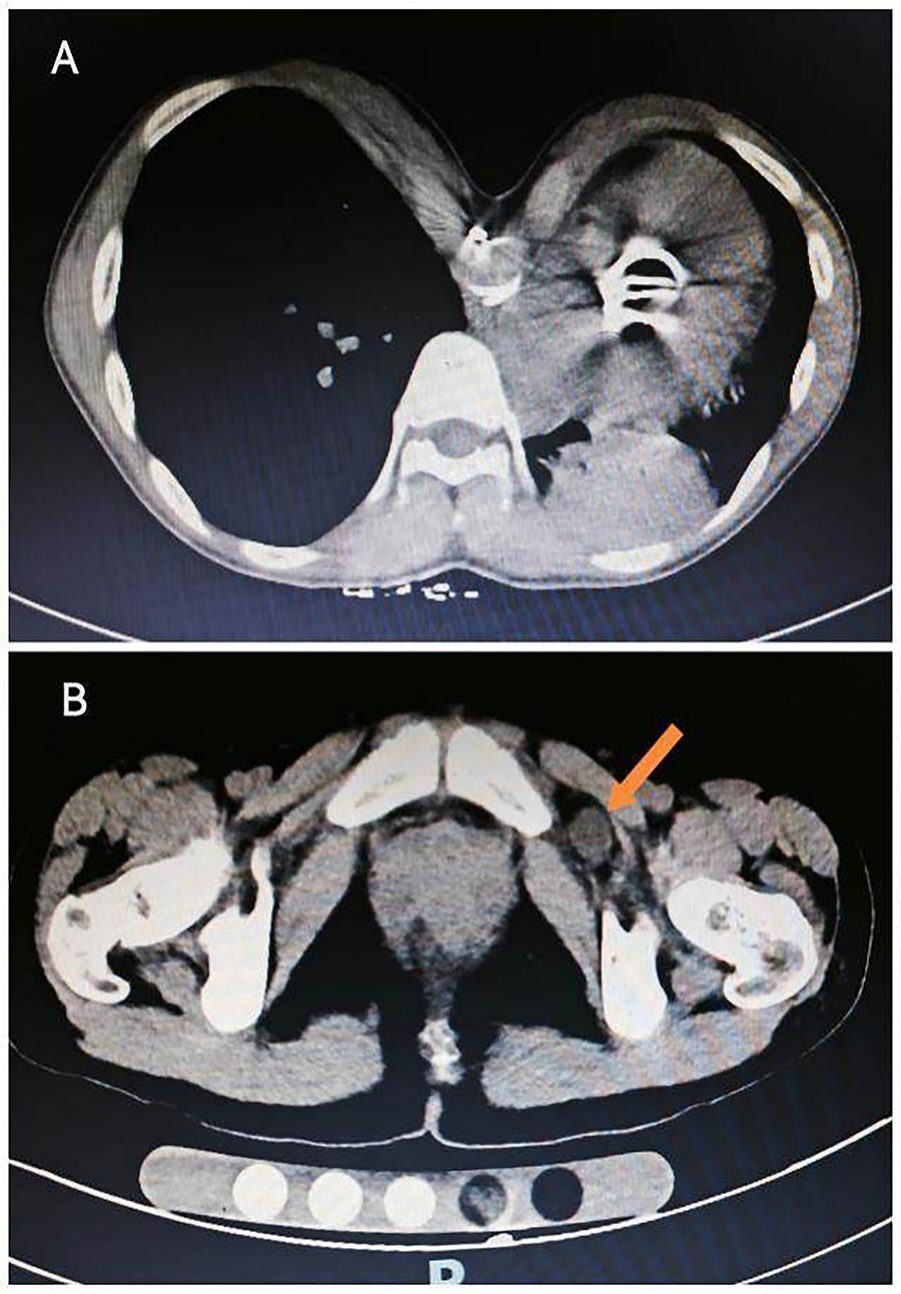
**(A)** chest CT showing cardiomegaly, post aortic valve replacement, pectus excavatum; **(B)** pelvic CT showing a left obturator hernia (the hernial contents between the external obturator muscle and the pectineus muscle are indicated by the orange arrow).

Upon admission, contrast-enhanced computed tomography (CT) of the abdomen and pelvis was performed to clarify the diagnosis, which revealed cardiomegaly, pectus excavatum (a classic skeletal manifestation of Marfan syndrome, Figure 1A), and a left-sided obturator hernia with herniation of extraperitoneal fat through the obturator canal (Figure 1B), without signs of intestinal incarceration or strangulation.

### 2.3 Therapeutic intervention

Based on the clinical manifestations, imaging findings, and past medical history, the admission diagnosis was established as Marfan syndrome complicated with left obturator hernia. Prior to surgery, comprehensive preoperative evaluations and laboratory tests were completed, including cardiac function assessment (electrocardiogram, echocardiogram) to ensure the patient's tolerance for anesthesia and surgery, as well as routine blood tests, coagulation function, and infectious disease screening to rule out surgical contraindications. The patient subsequently underwent laparoscopic preperitoneal inguinal hernia repair with mesh implantation, a minimally invasive approach preferred for its advantages of minimal tissue trauma, clear surgical field, and rapid postoperative recovery. Intraoperative exploration revealed a left-sided obturator hernia with a hernial ring diameter of approximately 1.0 cm. The bowel at the jejunoileal junction was compressed, exhibiting hyperemia and edema without evidence of necrosis. After peritoneal dissection, the Bogros and Retzius spaces were entered, with careful protection of vital blood vessels and triangular regions. A flat, lightweight, large-pore mesh measuring 15 × 10 cm was used to completely cover the myopectineal orifice, and it was fixed with absorbable tacks, followed by peritoneal suture. The anesthesia was effective, with an estimated blood loss of approximately 2 mL.

### 2.4 Follow-up and outcomes

Postoperatively, the patient received pain management, close monitoring of vital signs and wound conditions. No complications such as infection, bleeding, mesh rejection, or neurological symptoms occurred, and she achieved an uneventful recovery, being discharged smoothly on the 3rd postoperative day with regular follow-up arranged. At the 14-month follow-up, the patient recovered well with no hernia recurrence and continued oral warfarin anticoagulation therapy as prescribed.

Table 1 is the timeline that illustrates the clinical course from the patient's aortic valve replacement surgery, through the current admission for obturator hernia repair, to postoperative outpatient follow-up.

**Table 1:** From aortic valve replacement to postoperative follow-up after obturator hernia repair.

Timeline	Clinical Event
6 years prior to admission	Underwent aortic valve replacement surgery with uneventful postoperative recovery.
2 years prior to admission	Underwent cesarean section with uneventful postoperative recovery
Admission to the Emergency Department	Complaining of persistent severe pain in the left inguinal region for 6 h
-	Physical examination revealed a 2 cm × 2 cm localized swelling in the upper medial aspect of the left thigh, medial to the inguinal ligament. Left Howship-Romberg sign (+)
-	CT examination indicated pectus excavatum, cardiomegaly, and a left obturator hernia.
-	Laparoscopic preperitoneal inguinal hernia mesh repair was performed.
Within 3 days after surgery	Postoperative recovery was uneventful, and the patient was discharged on the 3rd postoperative day.
14 months postoperatively	The patient remained in good condition with no hernia recurrence.

## 3 Discussion

Obturator hernia is an extremely rare subtype of abdominal wall hernia, which accounts for only approximately 1% of all hernia cases. It usually happens in elderly female patients with a history of chronic increased intra-abdominal pressure or multiple pregnancies. It arises when increased intra-abdominal pressure leads to protrusion of abdominal viscera through the obturator canal. Clinically, patients typically present with nausea, vomiting, abdominal pain, and pain in the inguinal region or medial aspect of the thigh (5).

In our case report, we present a 26-years-old female patient with obturator hernia complicated by Marfan syndrome. It is rare in clinical practice because her age of onset is obviously younger than usual. In addition, Marfan syndrome is mostly associated with recurrent and incisional hernias, making cases complicated with obturator hernia even rarer, which provides a significant reference for the early identification and diagnosis of such conditions.

Marfan syndrome is an autosomal dominant hereditary connective tissue disorder. Its pathogenic mechanism is mainly caused by mutations in the FBN1 gene on chromosome 15, which leads to abnormal synthesis of fibrillin-1 and finally results in structural and functional impairments in multiple systems ranging from cardiovascular system to musculoskeletal system impaired (6). The patient of this case had classic clinical manifestations of Marfan syndrome, such as pectus excavatum, cardiomegaly, and a history of aortic valve replacement.

Mutations in the FBN1 gene can also weak the structure and elastic of fascia, ligament, and tendon tissues systemically, providing important pathological basis for happening of hernia. This can not only explain the high incidence of inguinal hernia and incisional hernia in patients with Marfan syndrome, but also offer a reasonable explanation for the mechanism of obturator hernia in our case. This is because the inherently weakened obturator fascia is more prone to be torn or develop defects with the effect of chronic or recurrent increased intra-abdominal pressure, such as previous pregnancy, chronic constipation and strenuous activity, which would promote the herniation of abdominal contents. In addition, the patient had undergone a cesarean section 2 years prior, which may have contributed to the weakening of abdominal wall connective tissues, creating a predisposing condition for hernia formation.

Up to now, literature search has shown that there is only one case report of Marfan syndrome complicated with obturator hernia worldwide (4). The patient in this case is a 26-year-old female. She ultimately underwent open laparotomy for intestinal obstruction secondary to obturator hernia. The two cases are highly similar in age of onset, gender and underlying disease characteristics between this case and our case, further supporting that Marfan syndrome may be a potential independent risk factor for the development of obturator hernia.

The mesh is primarily used to repair the defect. According to a review by Holm et al., mesh repair can effectively reduce the risk of hernia recurrence, with a recurrence rate of 2%, compared with 10% for simple sutured repair (7). Both flat and anatomical meshes are available. Although the long-term prognosis is comparable between the two types of mesh, anatomical meshes can alleviate patients' postoperative discomfort more effectively and provide a better quality of life in the short term (8). Unfortunately, due to resource constraints, only flat mesh was used in this study. Here, we chose a lightweight large-pore mesh measuring 15 × 10 cm and it was fixed with absorbable tacks, which can completely cover the myopectineal orifice. To prevent mesh migration and subsequent hernia recurrence, absorbable tacks were used to secure the mesh in place. However, mesh placement should be avoided in cases of intra-abdominal contamination to mitigate the risk of mesh infection (9).

Furthermore, no recurrences were reported following mesh repair in laparoscopic surgery, which indicates that laparoscopic surgery may also plays an important role in reducing the recurrence rate (7). Laparoscopic surgery offers multiple advantages, including faster recovery, smaller incisions, less blood loss, more aesthetic postoperative scars, a broad surgical field that facilitates operator manipulation, and shorter hospital stays for patients (10, 11). The laparoscopic approach encompasses two main techniques: the extraperitoneal approach (TEP) and the transperitoneal approach (TAPP). TEP avoids entering the peritoneal cavity, reducing the risk of intra-abdominal organ injury, and is associated with less postoperative pain and faster recovery. In contrast, TAPP provides a wider surgical field with clear anatomical landmarks and allows complete exploration of the abdominal cavity (12–14). In our case, TAPP was chosen to evaluate bowel viability, rule out contralateral hernias, and manage the deep and poorly exposed anatomical location of the obturator hernia. However, open surgery remains more prevalent in clinical practice. This may be attributed to the ability of open surgery to provide full abdominal exposure in emergency surgery, enabling thorough etiological exploration when the preoperative diagnosis is unclear (7).

The clinical significance of this case is to remind clinical doctors to broaden the thinking for the differential diagnosis of hernia complicated with connective tissue disease. Though inguinal hernia and incisional hernia are more common in patients with Marfan syndrome, we should pay more attention to the possibility of rare types of hernias, such as obturator hernia when patients present with acute intestinal obstruction, unexplained abdominal discomfort, or persistent pain in the medial thigh and knee, especially when the pain is exacerbated by hip flexion and internal rotation. Early implementations of targeted imaging examinations, such as pelvic CT or MRI, are supported to confirm the diagnosis, evaluate hernial contents and associated complications to avoid complications like strangulated intestinal obstruction and intestinal necrosis (15). It will help early interventions and improve prognosis if we attach more importance to such rare associations.

## Data Availability

The original contributions presented in the study are included in the article/Supplementary Material, further inquiries can be directed to the corresponding author.
